# Virological Characterization of the First 2 COVID-19 Patients Diagnosed in Italy: Phylogenetic Analysis, Virus Shedding Profile From Different Body Sites, and Antibody Response Kinetics

**DOI:** 10.1093/ofid/ofaa403

**Published:** 2020-09-02

**Authors:** Francesca Colavita, Daniele Lapa, Fabrizio Carletti, Eleonora Lalle, Francesco Messina, Martina Rueca, Giulia Matusali, Silvia Meschi, Licia Bordi, Patrizia Marsella, Emanuele Nicastri, Luisa Marchioni, Andrea Mariano, Laura Scorzolini, Tommaso Ascoli Bartoli, Antonino Di Caro, Giuseppe Ippolito, Maria Rosaria Capobianchi, Concetta Castilletti, INMI COVID-19 Laboratory Team and INMI COVID-19 Study Group, Isabella Abbate, Chiara Agrati, Loredana Aleo, Tonino Alonzi, Alessandra Amendola, Claudia Apollonio, Nicolina Arduini, Barbara Bartolini, Giulia Berno, Silvia Biancone, Mirella Biava, Angela Bibbò, Licia Bordi, Carla Brega, Marco Canali, Angela Cannas, Maria Rosaria Capobianchi, Fabrizio Carletti, Stefania Carrara, Rita Casetti, Concetta Castilletti, Roberta Chiappini, Lucia Ciafrone, Eleonora Cimini, Sabrina Coen, Francesca Colavita, Rossella Condello, Antonio Coppola, Silvia D’Arezzo, Antonino Di Caro, Stefania Di Filippo, Chiara Di Giuli, Lavinia Fabeni, Luisa Felici, Valeria Ferraioli, Federica Forbici, Anna Rosa Garbuglia, Emanuela Giombini, Caterina Gori, Silvia Graziano, Cesare Ernesto Maria Gruber, Daniele Khouri, Eleonora Lalle, Daniele Lapa, Barbara Leone, Patrizia Marsella, Chiara Massimino, Giulia Matusali, Antonio Mazzarelli, Silvia Meschi, Francesco Messina, Claudia Minosse, Claudia Montaldo, Stefania Neri, Carla Nisii, Elisabetta Petrivelli, Fabrizio Petroni, Elisa Petruccioli, Marina Pisciotta, Daniele Pizzi, Gianluca Prota, Fabrizio Raparelli, Gabriella Rozera, Martina Rueca, Rossella Sabatini, Silvia Sarti, Giuseppe Sberna, Roberta Sciamanna, Marina Selleri, Carla Selvaggi, Catia Sias, Chiara Stellitano, Antonietta Toffoletti, Silvia Truffa, Federica Turchi, Maria Beatrice Valli, Carolina Venditti, Tiziana Vescovo, Donatella Vincenti, Antonella Vulcano, Emma Zambelli, Maria Alessandra Abbonizio, Chiara Agrati, Fabrizio Albarello, Gioia Amadei, Alessandra Amendola, Mario Antonini, Raffaella Barbaro, Barbara Bartolini, Martina Benigni, Nazario Bevilacqua, Licia Bordi, Veronica Bordoni, Marta Branca, Paolo Campioni, Maria Rosaria Capobianchi, Cinzia Caporale, Ilaria Caravella, Fabrizio Carletti, Concetta Castilletti, Roberta Chiappini, Carmine Ciaralli, Francesca Colavita, Angela Corpolongo, Massimo Cristofaro, Salvatore Curiale, Alessandra D’Abramo, Cristina Dantimi, Alessia De Angelis, Giada De Angelis, Rachele Di Lorenzo, Federica Di Stefano, Federica Ferraro, Lorena Fiorentini, Andrea Frustaci, Paola Gallì, Gabriele Garotto, Maria Letizia Giancola, Filippo Giansante, Emanuela Giombini, Maria Cristina Greci, Giuseppe Ippolito, Eleonora Lalle, Simone Lanini, Daniele Lapa, Luciana Lepore, Andrea Lucia, Franco Lufrani, Manuela Macchione, Alessandra Marani, Luisa Marchioni, Andrea Mariano, Maria Cristina Marini, Micaela Maritti, Giulia Matusali, Silvia Meschi, Francesco Messina, Chiara Montaldo, Silvia Murachelli, Emanuele Nicastri, Roberto Noto, Claudia Palazzolo, Emanuele Pallini, Virgilio Passeri, Federico Pelliccioni, Antonella Petrecchia, Ada Petrone, Nicola Petrosillo, Elisa Pianura, Maria Pisciotta, Silvia Pittalis, Costanza Proietti, Vincenzo Puro, Gabriele Rinonapoli, Martina Rueca, Alessandra Sacchi, Francesco Sanasi, Carmen Santagata, Silvana Scarcia, Vincenzo Schininà, Paola Scognamiglio, Laura Scorzolini, Giulia Stazi, Francesco Vaia, Francesco Vairo, Maria Beatrice Valli

**Affiliations:** National Institute for Infectious Diseases “L. Spallanzani” IRCCS, Rome, Italy

**Keywords:** antibody response, COVID-19, Italy, phylogenesis, SARS-CoV-2, viral culture, virus shedding

## Abstract

**Background:**

The pathogenesis of severe acute respiratory syndrome coronavirus 2 (SARS-CoV-2) infection remains unclear. We report the detection of viral RNA from different anatomical districts and the antibody profile in the first 2 COVID-19 cases diagnosed in Italy.

**Methods:**

We tested for SARS-CoV-2 RNA clinical samples, either respiratory and nonrespiratory (ie, saliva, serum, urine, vomit, rectal, ocular, cutaneous, and cervico-vaginal swabs), longitudinally collected from both patients throughout the hospitalization. Serological analysis was carried out on serial serum samples to evaluate IgM, IgA, IgG, and neutralizing antibody levels.

**Results:**

SARS-CoV-2 RNA was detected since the early phase of illness, lasting over 2 weeks in both upper and lower respiratory tract samples. Virus isolate was obtained from acute respiratory samples, while no infectious virus was rescued from late respiratory samples with low viral RNA load, collected when serum antibodies had been developed. Several other specimens came back positive, including saliva, vomit, rectal, cutaneous, cervico-vaginal, and ocular swabs. IgM, IgA, and IgG were detected within the first week of diagnosis, with IgG appearing earlier and at higher titers. Neutralizing antibodies developed during the second week, reaching high titers 32 days after diagnosis.

**Conclusions:**

Our longitudinal analysis showed that SARS-CoV-2 RNA can be detected in different body samples, which may be associated with broad tropism and different spectra of clinical manifestations and modes of transmission. Profiling antibody response and neutralizing activity can assist in laboratory diagnosis and surveillance actions.

In January 2020, a novel coronavirus was identified as the cause of pneumonia cases, with the first cases reported in December 2019 in Wuhan City, Hubei Province of China [1, 2]. The new pathogen belongs to betacoronavirus genus lineage B, and due to its close phylogenetic relation to other bat severe acute respiratory syndrome (SARS)–like coronaviruses, it was named SARS coronavirus 2 (SARS-CoV-2). Initially linked to possible exposure to infected wildlife, human-to-human transmission was identified, and the outbreak rapidly spread to other parts of China and outside the country [2]. As of August 20, 2020, 22 431 929 coronavirus disease 2019 (COVID-19) cases (the illness caused by SARS-CoV-2), with 787_ _773 deaths have been reported worldwide [3].

Transmission is mainly through respiratory droplets, but other routes cannot be excluded and are under investigation, as SARS-CoV-2 was detected in several body fluids (ie, saliva, stool, ocular fluid) [1, 4–7]. Much still needs to be learned about this infection, and research is underway worldwide to better understand the clinical features and extent of interhuman transmission. A better knowledge of viral RNA shedding kinetics from different body districts could help us understand SARS-CoV-2 transmission and pathogenesis, supporting surveillance and clinical management. In addition, due to the current emergency context, very few data about the antibody response are available in the literature.

Here, we report the kinetics of viral RNA shedding from different body sites and the concomitant antibody profile (IgM, IgA, IgG, and neutralizing Ig) along the disease course in the first 2 COVID-19 confirmed cases reported in Italy and hospitalized at the National Institute for Infectious Diseases “Lazzaro Spallanzani” (INMI) in Rome.

## METHODS

### Clinical Samples

Clinical samples from the first 2 COVID-19 patients were longitudinally collected for diagnostic purposes starting from the first day of hospitalization (corresponding to day 1 from symptom onset [DSO], as declared by the patients at admission) up to DSO 32. These samples included upper (URT; ie, nasopharyngeal swab, nasal swab, throat swab) and lower (LRT; ie, sputum and bronchoalveolar lavage [BAL]) respiratory tract specimens and nonrespiratory specimens (ie, saliva, serum, urine, rectal swab, ocular swab, cervico-vaginal swab, cutaneous swab).

### Patient Consent Statement

The patients’ written consent was obtained. This study was approved by the INMI Ethical Board.

### Nucleic Acid Extraction and Molecular Tests

Viral RNA was extracted by QIAsymphony (QIAgen, Hilden, Germany), and real-time reverse transcription polymerase chain reaction (RT-PCR) targeting the E and RdRp viral genes was used to assess the presence of SARS-CoV-2 RNA [8]. Confirmation of diagnosis was performed by in-house RT-PCR targeting the viral membrane protein (M) gene, followed by Sanger sequencing (327 bp). Follow-up of the infection course was then performed using E gene real-time RT-PCR only. Other respiratory tract infections were investigated using multiplex nucleic acid testing (QIAstat-Dx Respiratory Panel, QIAgen, Hilden, Germany).

### Virus Isolation

Viral culture was performed in the BSL-3 laboratory, and clinical samples (ie, nasopharyngeal swab, sputum, BAL, and ocular swab) were diluted in MEM (Corning, New York, USA) plus viral inoculating broth (VIB) 1× containing antibiotics. The mixture was kept at room temperature for 1 hour and then inoculated on Vero E6 cells for 1 hour. Finally complete medium was replaced with MEM containing 2% FBS and 0.5× VIB. Cytopathic effect (CPE) appearance was observed by light microscope and the Cytation 5 reader (Biotek, Winooski, Vermont, USA). First, samples collected at diagnosis (nasopharyngeal swabs on both patients and sputum of Pt1) were immediately inoculated into the cell culture for isolation purposes. The follow-up samples (nasopharyngeal swabs, BAL, and ocular secretions) were stored at –80°C and never thawed before inoculation for viral culture, which was performed 3 months after sample collection.

### Next-Generation Sequencing and Bioinformatics Analysis

NGS was performed using the Ion Torrent (Thermo Fisher, Waltham, Massachusetts, USA) S5 platform as described in Capobianchi et al. [9]. Reads were de novo assembled, and consensus sequences were manually controlled and confirmed by Sanger sequencing.

The Bayesian phylogenetic tree was inferred using the Markov chain Monte Carlo (MCMC) approach in BEAST, version 1.10.4, with BEAGLE, version 2.1.2. To infer time-measured phylogenic analysis, the mutation model Hasegawa Kishino Yano (HKY) was used, which assumes the nucleotides have different frequencies into genome and, transitions and transversions occur at different rates [10.1007 / BF02101694]. Moreover, constant population size and strict clock model over time were imposed as coalescent priors for independent Monte Carlo Marchov Chain (MCMC) runs, as reported in previous study [10]. Chains were conducted for at least 100 × 106 generations with sampling every 10_ _000 steps and burn-in for 10 × 106 generations. The convergence of the MCMC was assessed by calculating for each parameter the Effective Sample Size (ESS) (accepted if the ESS > 250). A maximum clade credibility tree was obtained from the trees’ posterior distributions using Tree-Annotator, version 1.10.4.

### Serological Tests

Indirect immunofluorescence assay (IFA) was used to detect specific IgM, IgA, and IgG on slides prepared in-house with Vero E6 cells infected with SARS-CoV-2 isolate, as described elsewhere [11]. All sera were depleted of IgG using Eurosorb reagent (Euroimmun, Lubecca, Germany) and tested using 1:20 screening dilution with titration by limiting dilution. FITC-conjugated antihuman IgM, IgA, and IgG antibodies (Euroimmun, Lubecca, Germany) were used as secondary antibody and Evans Blue as cell counterstain.

For neutralizing antibody evaluation, sera were heat-inactivated, diluted 1:10, and titrated in duplicate in 2-fold dilutions. Equal volumes of 100 TCID50/well SARS-CoV-2 and serum dilutions were mixed and incubated at 37°C for 30 minutes. Subsequently, 96-well plates with subconfluent Vero E6 cells were incubated with 100 μL/well of virus-serum mixtures at 37°C, 5% CO2. Neutralizing antibody titers were calculated as the highest serum dilution not presenting CPE at day 6 postinfection.

## RESULTS

On January 29, 2020, 2 spouses, a 66-year-old woman (Patient 1 [Pt1]) and a 67-year-old man (Patient 2 [Pt2]) visiting Rome for vacation, were admitted at INMI as possible COVID-19 cases. Both patients arrived in Italy on January 23 from Wuhan, Hubei Province, China, and beginning January 28 presented relevant respiratory symptoms. Pt1 had a history of hypertension, whereas Pt2 had no comorbidities and milder illness at presentation, as recently reported [12].

**Figure 1.**
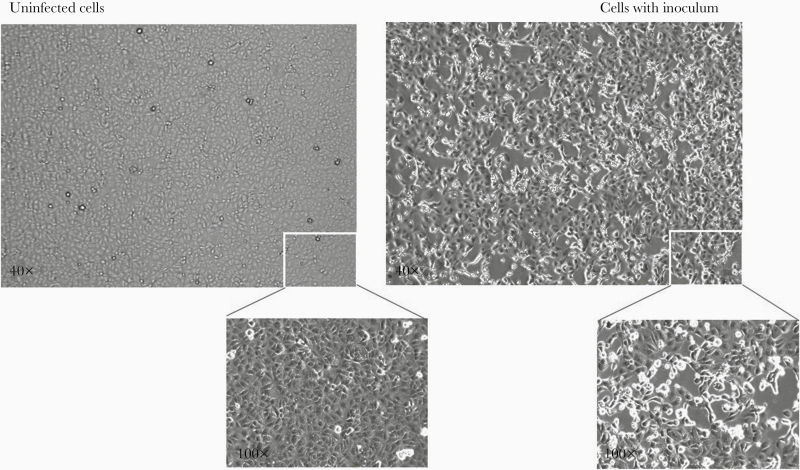
Severe acute respiratory syndrome coronavirus 2 (SARS-CoV-2) isolation in cell culture. Mock-infected Vero E6 cells (left) and cells inoculated with sputum from Pt1 (right) observed after 24 hours postseed. Magnification insets (100×) of selected regions are shown. Virus-induced cytopathic effect is evident in inoculated Vero E6 cells. Real-time reverse transcription polymerase chain reaction test on spent cell growth medium confirmed SARS-CoV-2 replication (inoculum cycle threshold [Ct] value = 16.73 vs 24 hours postinoculum Ct value = 8.15). Images captured by Cytation 5, Biotek.

Diagnosis of SARS-CoV-2 infection was confirmed by real-time RT-PCR on nasopharyngeal swab and sputum for Pt1 (cycle threshold [Ct]: 14.28 and 16.12, respectively) and on nasopharyngeal swab for Pt2 (Ct: 24.58), followed by viral M gene sequencing. Nasopharyngeal swabs at admission were negative for all other respiratory pathogens tested. Virus was isolated from Pt1 acute-phase sputum (named 2019-nCoV/Italy-INMI1) (Figure 1).

Both patients developed progressive respiratory failure on DSO 4 and required mechanical ventilation support in the intensive care unit (ICU) on DSO 6 for Pt2 and DSO 7 for Pt1. During their stay in the ICU, both patients received 3 days of lopinavir/ritonavir therapy, followed by intravenous administration of remdesivir for 13 days. At the time of writing, both patients were discharged.

Full-genome sequences of Pt1 were obtained by NGS from both virus isolate and clinical sample (nasopharyngeal swab). As described in Capobianchi et al. [9], the analysis of consensus sequences from the clinical sample showed 2 nonsynonymous changes with respect to the Wuhan-Hu-1 NCBI Reference Genome (accession number: MN908947.3), leading to change in Orf1a and in Orf3a. One additional synonymous substitution in Orf1a (A2269T) was detected in the isolate only [9]. The partial Pt2 sequence was very similar to the sequence of Pt1 and consistent with the full-genome sequence of the strain isolated by the national reference center (GISAID accession ID: EPI_ISL_412974) from Pt2’s nasopharyngeal swab [13].

**Figure 2.**
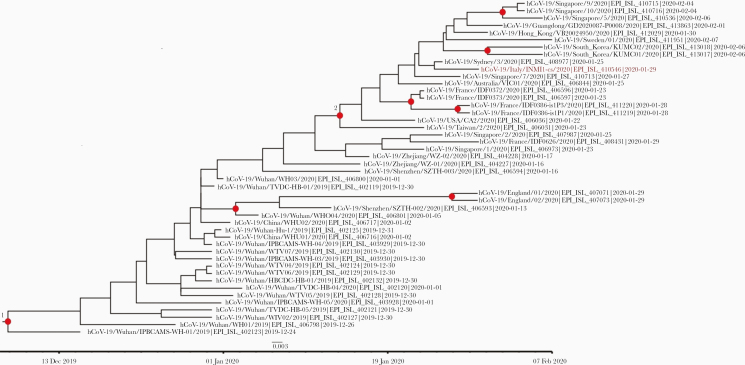
Estimated Bayesian maximum-clade-credibility tree of severe acute respiratory syndrome coronavirus 2 (SARS-CoV-2) whole-genome sequences. Red dots correspond to nodes with >85% posterior probability. The INMI-1 Pt1 sequence is highlighted in red. The nodes leading to the INMI-1 sequence segregation are shown in red. Chains were conducted for at least 100×10^6^ generations with sampling every 10 000 steps and burn-in 10×10^6^ generations. The convergence of the Markov chain Monte Carlo was assessed by calculating for each parameter the ESS (accepted if ESS > 250). A maximum clade credibility tree was obtained from the trees’ posterior distributions with the Tree-Annotator software, version 1.10.4.

Bayesian phylogenetic analysis places Pt1’s sequence (referred as INMI1) in the V clade, characterized by G251V substitution in the ORF3a gene, according to the NJ tree in the GISAID EpiCov portal. In this analysis, the origin of the entire clade V appears to date back to January 14 (95% HPD: January 5–23; node 2), which is highly consistent with the travel history of the patients from China. This analysis inferred a mutation frequency of 1.824 * 10–3 (95% HPD: 1.01 * 10–3 – 2.73 * 10–3) (Figure 2).

Several body fluids, including nonrespiratory specimens, were tested daily for SARS-CoV-2 RNA for 32 DSO. For Pt1, 148 samples, including 54 from the URT and LRT and 94 from other body sites (ie, saliva, vomit, serum, ocular swab, urine, rectal swab, cervico-vaginal swab, cutaneous swab) were tested. For Pt2, 119 samples were analyzed, including 48 from the URT and LRT and 71 from other body sites (ie, saliva, serum, ocular swab, urine, rectal swab, cutaneous swab).

**Figure 3.**
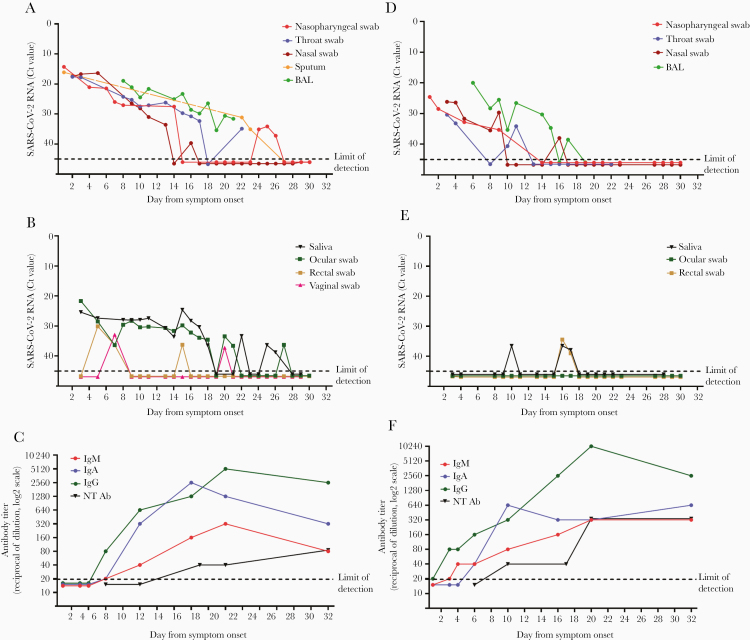
Kinetics of severe acute respiratory syndrome coronavirus 2 (SARS-CoV-2) RNA in different clinical samples and of antibody response in the first 2 coronavirus disease 2019 patients diagnosed in Italy. Viral RNA levels detected in respiratory tract secretions (A) and in non–respiratory tract samples (B) and antibody titers (C). Pt1 is shown on the left; pt2 is shown on the right. Antibody titers for IgM, IgG, IgA, and neutralizing antibodies (NT Ab) are expressed as the reciprocal of serum dilution and are shown on a log2 scale; viral RNA levels are expressed as cycle threshold values (Ct) of E gene amplification. Dashed lines represent the limits of detection of immunofluorescence assay (1:20 in (C) and (F)) and of real-time reverse transcription polymerase chain reaction (Ct: 45 in (A), (B), (D), and (E)).

The dynamics of viral RNA levels in different specimens are shown in Figure 3. Since DSO 1, for both patients, high viral loads were detected in respiratory samples. Compared with Pt2, Pt1 presented higher viral RNA levels in the URT at admission (difference of ~10 Ct) and during their hospitalization. During the progression of diseases, for both patients, specimens obtained from the LRT (ie, sputum and BAL) had higher SARS-CoV-2 RNA levels than those from the URT (Figure 3A and D). The last positive-testing result from a respiratory sample was at DSO 26 for Pt1 (nasopharyngeal swab) and DSO 17 for Pt2 (BAL). Viral culture was attempted on late follow-up respiratory samples collected at DSO 14 (nasopharyngeal swab, Ct: 27.5; and BAL, Ct: 23.3) and DSO 25 (nasopharyngeal swab, Ct: 34.1) from Pt1 and at DSO 14 (BAL, Ct: 30.3) from Pt2. No replication-competent virus was recovered from any of these samples. None of the urine samples from either patient tested positive for SARS-CoV-2 RNA. Serial saliva specimens came back negative for Pt2, but highly positive for Pt1 with a discontinuous and fluctuant trend of viral loads (Figure 3B and E). SARS-CoV-2 RNA was detected only in 1 out of 7 serum samples from Pt1 at DSO 5 (Ct: 35.5) and in none of the sera from Pt2 (not shown). All ocular swabs from Pt2 came back negative for SARS-CoV-2 (Figure 3E); on the contrary, viral RNA was detected in sequential ocular swabs collected from Pt1, who presented persistent bilateral conjunctivitis that was improved on 15 DSO and had resolved by DSO 20. In fact, as we have described elsewhere [14], SARS-CoV-2 RNA was detected in Pt1 ocular swabs starting from DSO 3 up to DSO 21 with declining viral RNA levels (Ct values from 21.66 to 36.56, respectively); a relapse was observed after 5 days of negative results, with a new positive result in the ocular swab sample collected at 27 DSO (Figure 3B). Notably, infectious virus was cultured from the first ocular sample, as detailed elsewere [13]. Rectal swabs were positive at DSO 5 (Ct: 30.10), 7 (Ct: 36.31), and 15 (Ct: 36.21) for Pt1 (Figure 3B) and at DSO 16 (Ct: 35.21) and 17 (Ct: 38.59) for Pt2 (Figure 3E). The unique vomit specimen collected at DSO 5 from Pt1 tested positive with a high RNA load (Ct: 19.49; not shown). SARS-CoV-2 RNA was detected also in cervico-vaginal swabs (Ct: 32.9 and 37.23) collected from Pt1 at DSO 7 and 20, respectively. Cutaneous swabs collected from the back of Pt2 were positive for SARS-CoV-2 at DSO 5 (Ct: 35,77) and negative at DSO 18; all cutaneous swabs available for Pt1 (at DSO 5 and 6) were negative (data not shown).

The kinetics of specific IgG, IgM, and IgA response was evaluated on serial serum samples in a time frame between DSO 1 and 32 (Figure 3C and F). For both patients, IgG was detected earlier and at higher titers than IgM and IgA, starting on DSO 6 for Pt1 and DSO 3 for Pt2. The titer of all antibody classes steadily increased since the second week of illness, mirroring an inverse trend toward decreasing levels of viral RNA in respiratory tract samples. Within the time frame considered for the serological investigation, a 4-fold increase in IgM titers, a 6-fold increase in IgA, and a 8-fold increase in IgG were observed in Pt1; for Pt2, a 4-fold increase in IgM and IgA titers and a 9-fold increase in IgG titers were found. Both patients developed neutralizing antibodies, which were first detected at DSO 10 for Pt2 and DSO 17 for Pt1. The neutralization titer steadily increased in both patients, reaching 1:320 in Pt1 and 1:80 in Pt2 at DSO 32. As shown in Figure 3, an earlier and more robust seroconversion occurred in Pt2 in comparison with Pt1; inversely, along the entire disease course, the virus RNA levels in virtually all body sites were lower in Pt2 as compared with Pt1.

## CONCLUSIONS

Here, we report virological and serological characterization of the first 2 COVID-19 cases diagnosed in Italy during the 2020 pandemic. The 2 patients traveled from Whuan, China, to Italy on January 23, developed symptoms in Rome on January 28, and were hospitalized the following day. A detailed description of the clinical presentation has been published [12].

The patients harbored the same virus strain, clustering with clade V, characterized by a nonsynonymous mutation in the ORF3a gene (G251V), according to the GISAID EpiCov portal [9, 13]. Bayesian phylogenetic analysis is consistent with the plausible date of exposure that presumably occurred in China before the start of their travel.

Sequential sampling in a wide range of body fluids was performed to monitor viral dissemination and shedding as well as antibody kinetics throughout the illness.

SARS-CoV-2 RNA was detected both in URT and LRT samples since the initial phase of disease. Similarly to what was observed in MERS and SARS patients, our analysis showed that LRT samples presented higher levels of SARS-CoV-2 RNA than those in paired samples from the URT [15–17]. The results are consistent with the expression of the candidate SARS-CoV-2 cell entry receptor, human angiotensin-converting enzyme 2 (ACE2), which is found primarily in the LRT [18, 19].

Nevertheless, the presence of high levels of viral RNA in the URT samples during the early phase of illness, coupled with the isolation of infectious virus obtained by others groups [20] and by us on different patients (authors’ unpublished data), strongly suggests a high potential for SARS-CoV-2 transmission [20, 21]. Duration of viral shedding in respiratory samples was 26 DSO for Pt1 and 17 for Pt2, in line with observations reporting 20 days as the median shedding duration for survivors [22, 23]. Live virus was isolated from the respiratory samples collected at presentation from the 2 patients [13]. In line with previous reports [20, 23], despite numerous attempts in Vero E6 cell culture, no replication-competent virus was recovered from later respiratory samples when antibodies were detected. Several factors, including suboptimal sensitivity of the virus culture system especially for low–viral load samples and storage at –80°C, as well as the presence of antibodies against SARS-CoV-2, may have contributed to unsuccessful virus culture attempts. However, the difficulty isolating SARS-CoV-2 from late samples supports the idea that despite the long duration of viral RNA shedding, the transmission of the infection is likely limited to early infection, when the viral load is high and antibody response has not yet been developed [23]. Shorter duration of viral shedding was observed for nonrespiratory samples, which showed lower viral loads since the early phase of illness and a more discontinuous trend.

Among the nonrespiratory samples, saliva was positive from the early to late phases of disease (up to DSO 26) for Pt1, supporting the idea of transmission via saliva droplets [4]. Stool represents another specimen of clinical and epidemiological interest, and stool sample testing for follow-up monitoring and patient discharge has been suggested. In fact, the presence of SARS-CoV-2 RNA in fecal samples has been reported even after viral clearance from the respiratory tract [5, 24], and our results on rectal swabs partially support these data from the literature, as fluctuant positivity was found during illness. However, to our knowledge there have been no reports of fecal–oral transmission yet, and this issue is still under debate, although several authors have provided data suggesting that, at least in some cases, the gastrointestinal tract may harbor SARS-CoV-2 replication [25, 26]. The presence of SARS-CoV-2 in blood is still controversial: Some reports on COVID-19 patients found no viral RNA, while in other studies occasional (10%) positivity was reported, possibly associated with severe manifestations [27–31]. In this study, only 1 serum sample from Pt1 tested positive for SARS-CoV-2 RNA with high real-time RT-PCR Ct values; this was collected at DSO 5 and corresponded to worsening of the clinical picture and ICU admission. However, the detection of low-level viral genome fragments in blood is not to be taken as definitive evidence of bloodstream dissemination of the virus; from our and other existing data, it seems that blood does not play a major role in virus transmission [23, 28, 32].

We did not find viral RNA in urine samples from the patients. However, urinary shedding of viral RNA has been occasionally reported with evidence of renal tropism [28, 31–33], and infectious virus was cultured from the urine of a COVID-19 case in China [34]. The limited number of patients included in our study may account for the apparent discrepancy and does not allow us to establish a definitive role of urinary shedding in viral diagnosis. Attention to the possible involvement of conjunctiva, either as the site of virus entry or a source of contagion, has been suggested [35, 36]. Ocular samples collected from Pt1 (who presented conjunctivitis at admission and up to DSO 20) were positive for SARS-CoV-2 RNA from the very early phase of infection up to DSO 27. Surprisingly, as we have described elsewhere [14], infectious virus was cultured from the first acute ocular sample, supporting the evidence of persistent sustained viral replication in conjunctiva and viral shedding from this site [14]. No virus detection was observed for Pt2, who did not present any ocular symptoms. These findings indicate that contact with conjunctival secretion from COVID-19 patients with ocular symptoms may represent a potential risk of infection; therefore, eye protection represents an important measure to prevent virus transmission especially in health care settings. SARS-CoV-2 RNA was recovered from several additional nonrespiratory samples. Although at low levels, we found positive for viral RNA a cutaneous swab from Pt2, and vomit samples and cervico-vaginal swabs from Pt1. These findings are thus far unique [37] and need to be confirmed in further studies in order to define the transmission potential linked to this wide RNA shedding.

To date, knowledge on the antibody response during SARS-CoV-2 infection is limited. We monitored the kinetics of IgM, IgA, IgG, and neutralizing antibodies in the 2 patients using IFA based on whole virus in serial samples collected during hospitalization. In line with other reports on COVID-19 cases, we observed seroconversion for all antibody classes within the first week after diagnosis, which corresponds to the date of symptom onset based on the recorded anamnestic data provided by the patients [31, 38, 39]. Surprisingly, in both patients IgG was detected at 3 (Pt1) and 6 (Pt2) DSO at high titers, when IgM and IgA were still low or undetectable [40, 41]. We cannot exclude the occurrence of a pauci-symptomatic phase that may have prolonged the effective time lapse from the initial infection and IgG appearance. The early appearance of high-titer IgG in contrast to IgM could be also due to an anamnestic response to past infection with other endemic coronaviruses, as previously reported [20, 42].

Increasing antibody levels were observed during the second week, with high titers of IgG and IgA. In addition, in accordance with earlier findings, both patients developed neutralizing antibodies during the second week of illness, reaching high levels at DSO 32 [31]. IgA is predominantly present in mucosal tissues, including the URT, providing the first line of defense in mucosal immunity. As shown in this study and others, detecting seroconversion of IgA as well as IgG and IgM can be useful to fully evaluate the humoral response in COVID-19 cases [38, 39].

Although our study examined 2 patients, which represents a limitation of the present results, the description of virus dynamics based on daily monitoring of both virological and serological aspects during the course of disease can give important insight into the pathogenesis and host response. Overall, the results show that, on one side, SARS-CoV-2 shedding and its duration may involve several body sites and may be associated on different. spectrum of clinical manifestation (such as conjuntivitis). Further studies are needed for a better understanding of this aspect, which is important to inform clinical management and public health decision-making. On the other side, the detection and profile of specific antibodies can assist with diagnosis, provide valuable information for screening of suspect cases (including in subclinical cases), and evaluate the disease course. Furthermore, the evaluation of antibody response will be crucial for surveillance and epidemiological studies of this novel disease and may be informative in vaccine development for SARS-CoV-2. Further investigation should clarify the level and duration of protection following infection.
